# Characterization of Recombinant Antimicrobial Peptide BMGlv2 Heterologously Expressed in *Trichoderma reesei*

**DOI:** 10.3390/ijms231810291

**Published:** 2022-09-07

**Authors:** Qingping Liang, Linyuan Cao, Changliang Zhu, Qing Kong, Han Sun, Fang Zhang, Haijin Mou, Zhemin Liu

**Affiliations:** College of Food Science and Engineering, Ocean University of China, Qingdao 266003, China

**Keywords:** antimicrobial peptide, BMGlv2, *Trichoderma reesei*, heterologous expression, piglet diarrhea

## Abstract

Antimicrobial peptides (AMPs) serve as alternative candidates for antibiotics and have attracted the attention of a wide range of industries for various purposes, including the prevention and treatment of piglet diarrhea in the swine industry. *Escherichia coli*, *Salmonella*, and *Clostridium perfringens* are the most common pathogens causing piglet diarrhea. In this study, the antimicrobial peptide gloverin2 (BMGlv2), derived from *Bombyx mandarina*, was explored to determine the efficient prevention effect on bacterial piglet diarrhea. BMGlv2 was heterologously expressed in *Trichoderma reesei* Tu6, and its antimicrobial properties against the three bacteria were characterized. The results showed that the minimum inhibitory concentrations of the peptide against *E. coli* ATCC 25922, *S. derby* ATCC 13076, and *C. perfringens* CVCC 2032 were 43.75, 43.75, and 21.86 μg/mL, respectively. The antimicrobial activity of BMGlv2 was not severely affected by high temperature, salt ions, and digestive enzymes. It had low hemolytic activity against rabbit red blood cells, indicating its safety for use as a feed additive. Furthermore, the measurements of the leakage of bacterial cell contents and scanning electron microscopy of *C. perfringens* CVCC 2032 indicated that BMGlv2 exerted antimicrobial activity by destroying the cell membrane. Overall, this study showed the heterologous expression of the antimicrobial peptide BMGlv2 in *T. reesei* and verified its antimicrobial properties against three common pathogenic bacteria associated with piglet diarrhea, which can provide a reference for the applications of AMPs as an alternative product in industrial agriculture.

## 1. Introduction

Due to the inappropriate management of sanitation and feed, piglets are prone to diarrhea after weaning and being transferred to nursing homes. The mortality rate due to piglet diarrhea may even be as high as 60%, resulting in heavy economic losses in the pig breeding industry [1,2,3]. Piglet bacterial diarrhea is considered one of the common forms of diarrhea, with the main pathogenic bacteria including *E. coli* [4], *Salmonella* [5], and *C. perfringens* [6]. These pathogenic bacteria usually colonize the intestines of piglets by binding to the receptors of the intestinal epithelial cells via their virulence traits, including adhesins, flagella, and biofilm-related proteins, and the bacteria then secrete toxins or depend on other virulence factors to exhibit the pathogenicity, leading to diarrhea [7,8]. Piglet bacterial diarrhea is usually manifested as piglet yellow-white dysentery, piglet paratyphoid fever, and piglet red dysentery, which are caused by *E. coli*, *Salmonella*, and *C. perfringens*, respectively [9]. The presence of pathogenic bacteria in piglet intestines is likely to increase the efficiency of other factors and further aggravate piglet diarrhea [10,11]. In addition, persistent diarrhea results in dehydration and malnutrition and causes huge economic losses in the pig industry [4].

The use of antibiotics to treat bacterial diarrhea inhibits the growth of special pathogens and has been common in piglet husbandry in the past few years [12]. For example, tetracycline, bacitracin, and enrofloxacin are available as anti-*C. perfringens* agents [13,14], and cefalexin administration is effective for *Salmonella* infection [5]. However, the overuse of antibiotics has resulted in the continuous emergence of drug-resistant strains and widespread drug resistance, which has gained increased attention from researchers [15,16]. Moreover, the European Union and China have introduced rules prohibiting the use of antibiotics in swine feed [17,18]. Therefore, there is an urgent need to seek in-feed antibiotic alternatives for the inhibition of pathogenic bacteria related to piglet husbandry [19].

Antimicrobial peptides (AMPs), potential substitutes for antibiotics, exhibit strong antimicrobial activity and have a wide range of sources [20,21,22,23]. Most of the reported AMPs have been confirmed to have extensive antimicrobial activity against Gram-positive and Gram-negative bacteria and even fungi, showing great potential value when applied in agricultural breeding [24,25]. Among them, microcin J25, pediocin A, plectasin, and lactoferrin have been proved to have a good bactericidal effect on common pathogenic bacteria of animals, such as *E. coli* and *Salmonella* [26]. However, there are rarely studies that report the antimicrobial activity of AMPs against *C. perfringens*. In addition, the industrialized application of AMPs to treat bacterial diarrhea in piglets is still minimal. Considering these problems, it is of great significance to study the antimicrobial properties of AMPs as potential feed additives in piglet husbandry.

Currently, the most widely used AMPs as feed additives in agriculture are insect AMPs belonging to the cecropin family, which have a unique structure and are rich in specific amino acids [27,28]. Gloverin, one of the glycine-rich AMPs, has been reported to possess strong antimicrobial activity against *E. coli* and *Pseudomonas putida* at the concentration of 10 μM [29,30,31]. However, the application of gloverin for the treatment of piglet diarrhea, especially that caused by *C. perfringens*, is deficient. Furthermore, to meet the requirements of industrial production as a feed additive, gloverin needs to be produced through microbial heterologous expression. Heterologous expression using a safe host is necessary for its application in aquaculture. *Trichoderma reesei* has been gradually introduced as a generally recognized as safe (GRAS) strain for recombinant protein expression, with strong expression promoters, superior protein secretion capabilities, and a low fermentation cost, providing a more valuable option for industrial protein production [32]. Furthermore, *T. reesei* has native cellulase activity that helps to degrade cellulose in the feedstuff [33], which is beneficial for the absorption of feed nutrients by animals.

In this study, gloverin derived from *Bombyx mandarina*, BMGlv2, was expressed in *T. reesei* Tu6. Three bacteria possibly associated with piglet diarrhea, *E. coli* ATCC 25922, *S. derby* ATCC 13076, and *C. perfringens* CVCC 2032, were used to determine the antimicrobial activity of BMGlv2. Thermostability and other biochemical characteristics were also accessed. Furthermore, the inhibition mechanism of BMGlv2 against *C. perfringens* was elucidated in terms of bacterial cell content leakage and cell membrane damage. 

## 2. Results

### 2.1. Construction of Recombinant Expression Vector PCBHG-BMGlv2

The amino acid sequences of BMGlv2 from *B. mandarina* were obtained from the NCBI database (accession number ANS56421.1), and the sequences of the gene encoding the mature protein were from the GenBank with the accession number of OL331022. The synthetic gene fragment of BMGlv2 was amplified by a PCR and verified using nucleic acid electrophoresis, and the position of the band showed that it was almost consistent with the theoretical DNA fragment length (539 bp) (Figure S1A). The purified gene fragment was inserted and ligated to the expression vector of *T. reesei* Tu6 PCBHG. After transferring the constructed plasmid into *E. coli* DH5α, the transformants grown were selected and verified using a colony PCR (Figure S1B). The recombinant expression vector PCBHG-BMGlv2 was obtained after the verification of positive transformants by sequencing. 

### 2.2. Expression and Purification of BMGlv2 in T. reesei

PCBHG-BMGlv2 was transferred into *T. reesei* Tu6, and the genomes of the grown *T. reesei* transformants were extracted for positive screening using PCR. The preliminary results showed that the target gene sequence existed in the screened transformant genomes (Figure S1C). The screened transformants were verified by sequencing, and one of them was transferred to a liquid medium for fermentation and expression. 

The recombinant BMGlv2 was purified to further evaluate its properties. The initial antimicrobial activity and yield of BMGlv2 are summarized in Table 1. After purification, the activity and specific activity of BMGlv2 increased to 292 AU/mL and 2555 AU/mg, respectively, compared with the fermented product. Additionally, the yield of BMGlv2 reached 54.5% in the process of purification. Overall, the purified BMGlv2 was obtained and the increase in its activity was achieved.

### 2.3. Antimicrobial Activity of BMGlv2

The antimicrobial activity of purified BMGlv2 was measured against three common bacteria causing piglet diarrhea, and the results demonstrated that BMGlv2 showed inhibitory effects on all three tested bacteria, with MIC values of 43.75, 43.75, and 21.86 μg/mL for *E. coli* ATCC 25922, *S. derby* ATCC 13076, and *C. perfringens* CVCC 2032, respectively (Table 2). The MBC values of BMGlv2 were the same as the MIC values (Table 2), indicating that BMGlv2 completely inhibits bacterial growth at a sufficient concentration.

### 2.4. Temperature and Salt Sensitivity

**Figure 1:**
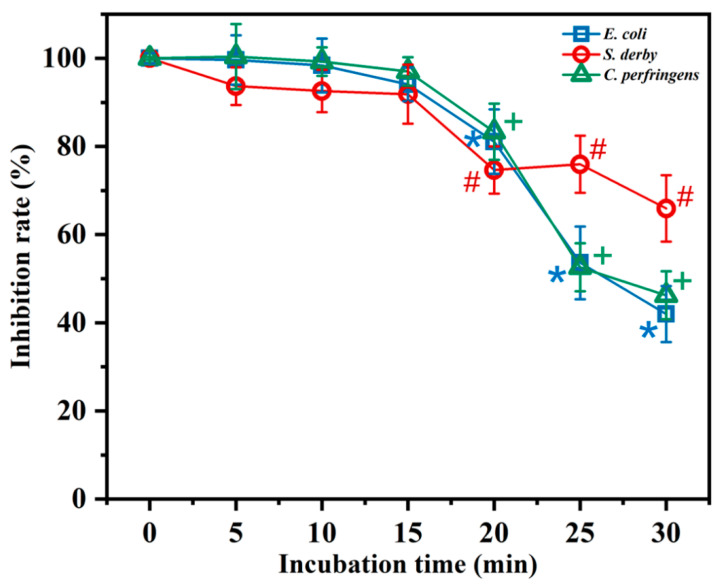
Effect of thermostability on the antimicrobial activity of BMGlv2 against *E. coli* ATCC 25922, *S. derby* ATCC 13076, and *C. perfringens* CVCC 2032. BMGlv2 at the concentrations of its MICs against the three tested bacteria was heated in boiling water at different incubation times (5, 10, 15, 20, 25, and 30 min), and the inhibition rate of BMGlv2 against the three tested bacteria was measured and calculated. *, #, and + represent the significant differences among different incubation times of BMGlv2 compared with the zero-time point of the inhibition rate of *E. coli* ATCC 25922, *S. derby* ATCC 13076, and *C. perfringens* CVCC 2032, respectively. Statistical analysis was calculated using variance (*t*-test) with IBM SPSS Statistics 26 software (*p* < 0.05), and the results were given as the mean ± standard deviation (SD).

To understand the effect of different environments on the antimicrobial activity of BMGlv2, thermostability and salt sensitivity were measured. The thermostability was determined after the incubation of BMGlv2 in boiling water at different times (5, 10, 15, 20, 25, and 30 min). The results indicated that the high-temperature treatment did not affect the antimicrobial activity of BMGlv2 against the three tested bacteria within 15 min of heating; however, heating for 30 min decreased the activity by 58.0%, 34.1%, and 53.8% against *E. coli* ATCC 25922, *S. derby* ATCC 13076, and *C. perfringens* CVCC 2032, respectively (Figure 1). Furthermore, the inactivation of BMGlv2 on *S. derby* ATCC 13076 was the least obvious, and the antimicrobial activity of BMGlv2 showed a similar trend against *E. coli* ATCC 25922 and *C. perfringens* CVCC 2032 after heating for different lengths of time. 

The influence of monovalent (150 mM NaCl, 4.5 mM KCl, and 6.0 μM NH_4_Cl), bivalent (8.0 μM ZnCl_2_ and 1.0 mM MgCl_2_), and trivalent (4.0 μM FeCl_3_) salts at their physiological concentrations on the antimicrobial activity of BMGlv2 was examined. The results showed that the MICs of BMGlv2 for the three tested bacteria were not affected or were partially affected (Table 3). Among them, *S. derby* ATCC 13076 showed no change in MICs, whereas the MICs of the other two bacteria decreased to a certain extent. Interestingly, Na^+^, Zn^2+^, and Fe^3+^, which have different valence cations, promoted the antimicrobial activity of BMGlv2 against *E. coli* ATCC 25922 and *C. perfringens* CVCC 2032, whereas K^+^ did not affect the antimicrobial activity against all three tested bacteria.

### 2.5. Resistance to Digestive Enzymes

**Figure 2:**
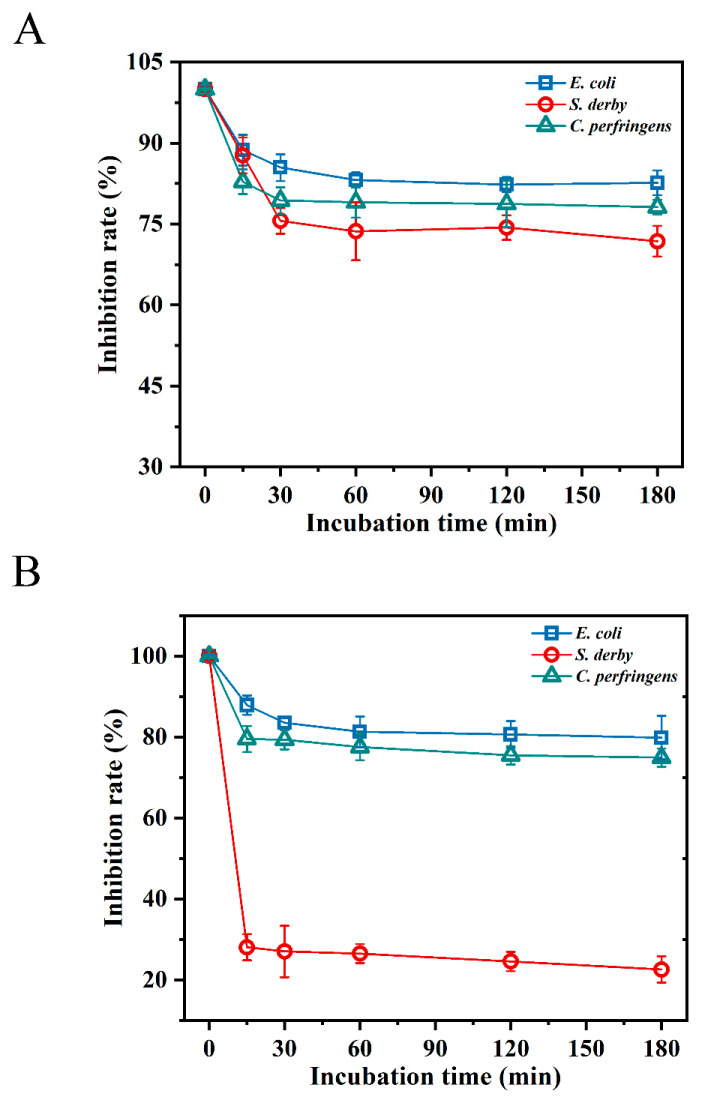
Effect of digestive enzymes on the antimicrobial activity of BMGlv2 against *E. coli* ATCC 25922, *S. derby* ATCC 13076, and *C. perfringens* CVCC 2032. (**A**) The effect of pepsin on the antimicrobial activity of BMGlv2. (**B**) Effect of trypsin on the antimicrobial activity of BMGlv2. Results were given as the mean ± SD.

According to the results of the digestive enzyme sensitivity analysis, BMGlv2 was more adaptable to the pepsin environment (pH 2.0), and its sensitivity to pepsin was weak. After 3 h of pepsin treatment, it still maintained an 82.6%, 71.8%, and 78.1% inhibition rate against *E. coli* ATCC 25922, *S. derby* ATCC 13076, and *C. perfringens* CVCC 2032, respectively (Figure 2A). However, after treatment with trypsin (pH 8.0) for 15 min, the inhibition rate of BMGlv2 against *S. derby* ATCC 13076 decreased to 28.1%, whereas under the same treatment, the inhibition rate against *E. coli* ATCC 25922 and *C. perfringens* CVCC 2032 was less affected, with inhibition rates of 87.9% and 79.5%, respectively (Figure 2B).

### 2.6. Bactericidal Kinetic Assays

**Figure 3:**
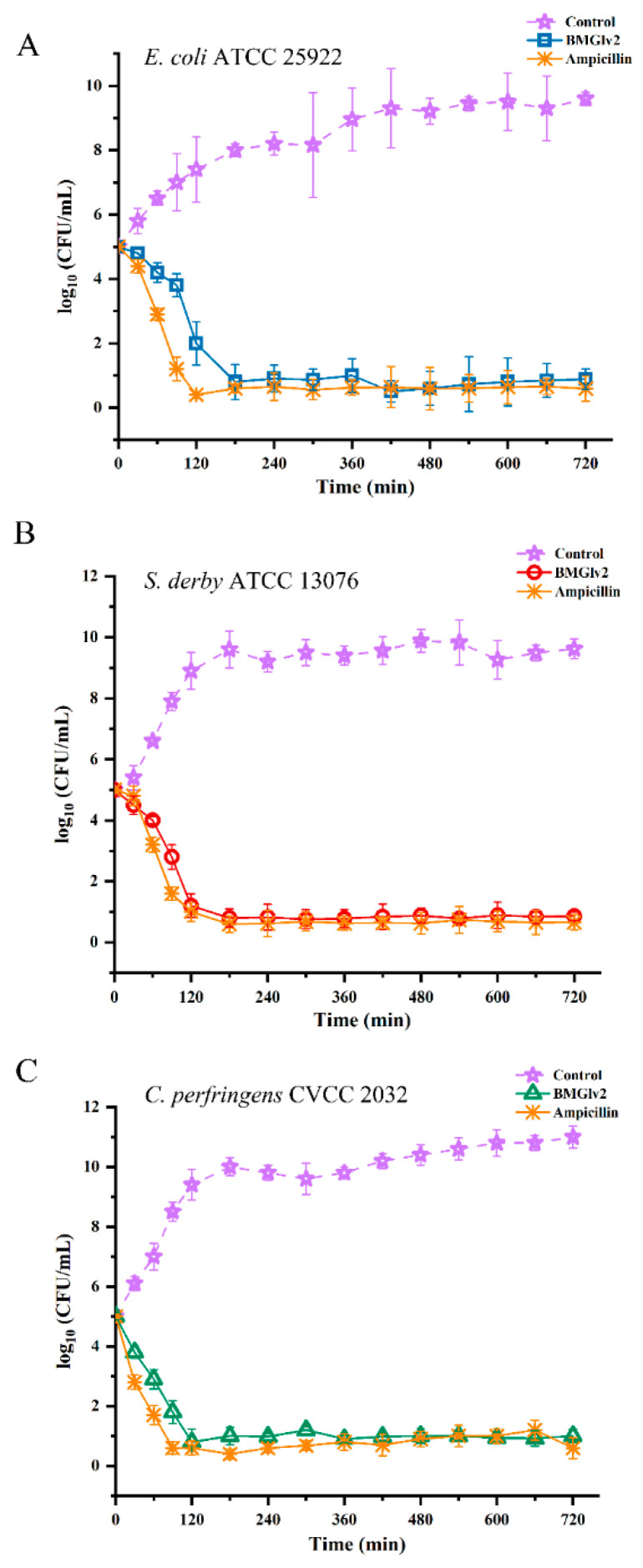
Time-kill curves of BMGlv2. (**A**) Time-kill curves of BMGlv2 against *E. coli* ATCC 25922. (**B**) Time-kill curves of BMGlv2 against *S. derby* ATCC 13076. (**C**) Time-kill curves of BMGlv2 against *C. perfringens* CVCC 2032. Results were given as the mean ± SD.

The growth kinetics of BMGlv2 were determined to evaluate the pharmacodynamics of the bacteria tested in this study. After a treatment of 2 h with BMGlv2, the counts of *E. coli* ATCC 25922, *S. derby* ATCC 13076, and *C. perfringens* CVCC 2032 decreased from 10^5^ CFU/mL to 10^2^ CFU/mL, 10^1.7^ CFU/mL, and 10^0.8^ CFU/mL, respectively, whereas the control groups of the three bacteria increased from 10^5^ CFU/mL to 10^7.3^ CFU/mL, 10^9^ CFU/mL, and 10^9.5^ CFU/mL, respectively (Figure 3A–C). The results showed that BMGlv2 exerted an antibacterial effect rapidly, especially for *C. perfringens* CVCC 2032. Furthermore, ampicillin had a more significant inhibitory effect on the three bacteria within 3 h of the treatment time, whereas BMGlv2 exhibited a comparable kinetic effect in the following time (Figure 3A–C).

### 2.7. Hemolytic Activity Assays

**Figure 4:**
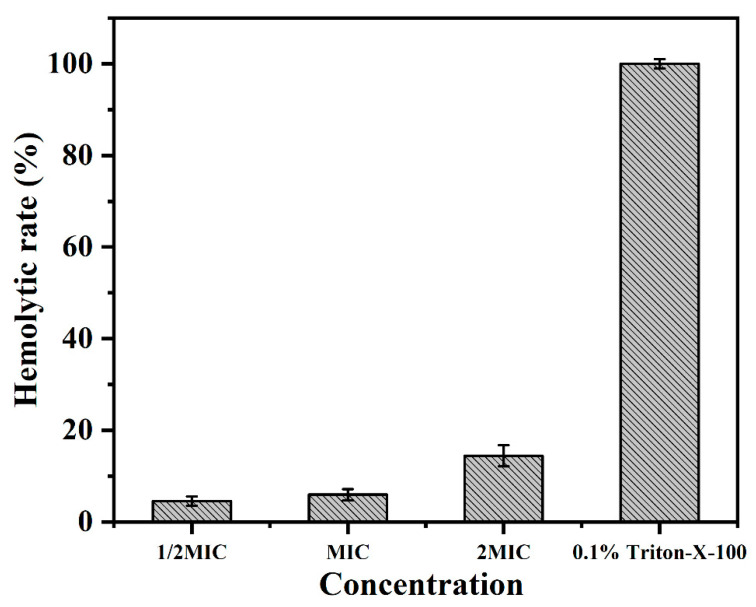
Hemolytic activity of BMGlv2 against rRBCs. For BMGlv2 against *E. coli* ATCC, MIC was 25922, with a value of 43.75 μg/mL. The hemolysis values (%) were defined according to the equation: (OD570 of BMGlv2 solution − OD570 of PBS)/(OD570 of 0.1% Triton X-100 − OD570 of PBS) × 100%. Triton X-100 acted as the positive control, PBS acted as the negative control. Results were given as the mean ± SD.

The hemolytic activity of BMGlv2 was studied by evaluating its ability to lyse rRBCs at different concentrations (1/2 MIC, 1 MIC, and 2 MIC against *E. coli* ATCC 25922). The results showed that the hemolysis values (%) increased with the increase in the concentration of BMGlv2. BMGlv2 did not exhibit significant hemolytic activity against rRBCs, even at the highest measured concentration of 87.5 μg/mL (Figure 4), indicating that BMGlv2 had little hemolytic activity at its effective concentrations and it is safe to use on the rRBCs.

### 2.8. Action Mechanism of BMGlv2 against C. perfringens

**Figure 5:**
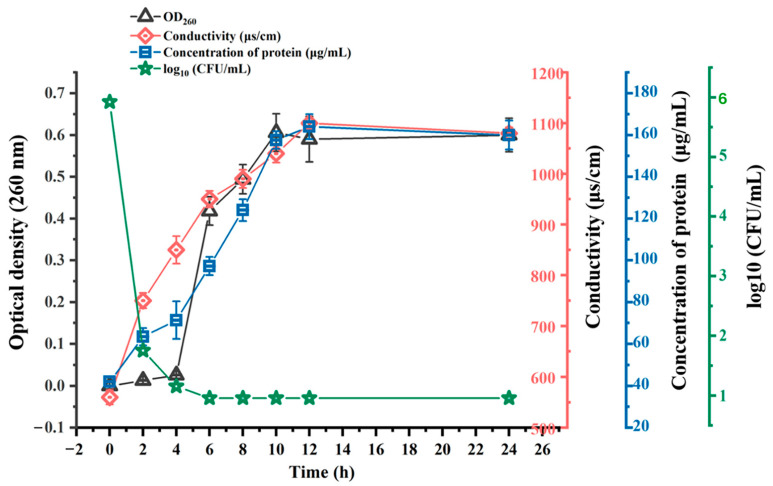
Mechanisms of action of BMGlv2 against *C. perfringens* CVCC 2032 including DNA leakage, protein leakage, alteration of conductivity, and bacterial counts. Results were given as the mean ± SD.

The leakage of nucleic acids in *C. perfringens* CVCC 2032 cells was determined by measuring the absorbance at 260 nm at different times. With the inhibition of BMGlv2 at the MIC, the nucleic acid content in the bacterial culture medium gradually increased (Figure 5). The OD_260 nm_ values changed from 0.01 to 0.41 within 6 h after treatment with BMGlv2 and remained almost unchanged for the next 6 h. The fastest time of nucleic acid leakage was 0–2 h, and the OD_260 nm_ values varied from 0.01 to 0.15.

To better understand the degree of damage to the cell membrane of *C. perfringens* CVCC 2032, its extracellular protein content was determined using the Coomassie brilliant blue method. The results showed that as the treatment time increased, the protein content of the bacterial supernatant gradually increased and tended to level off. After 8 h of being in culture with BMGlv2 in the bacterial liquid, the protein content reached the highest level, increasing from the initial 0.06 to 0.16 mg/mL (Figure 5), which indicated that the bacterial cell membrane was gradually destroyed.

The alteration in conductivity can also reflect the degree of leakage of the bacterial cell content that resulted from cell membrane damage. Under the intervention of BMGlv2 at the MIC, the conductivity of the bacterial solution exhibited an upward trend (Figure 5), which increased from 750 μs/cm at 0 h to 1100 μs/cm at 12 h, then tended to stabilize.

The visible colony counts of *C. perfringens* CVCC 2032 were determined at different treatment times of BMGlv2. The results demonstrated that the bacterial colonies decreased sharply at 4 h, indicating that BMGlv2 rapidly exerted its antimicrobial activity (Figure 5).

**Figure 6:**
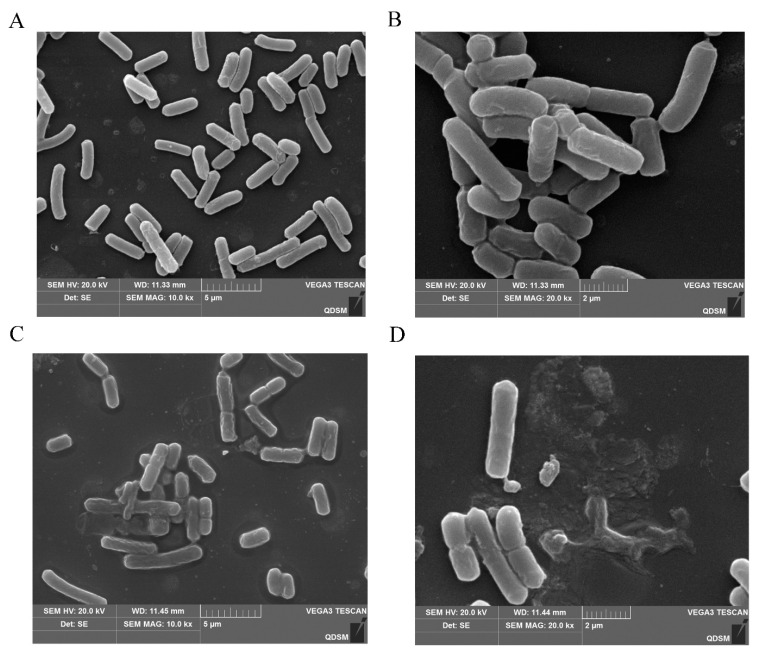
SEM micrographs of *C. perfringens* CVCC 2032 treated with BMGlv2. (**A**,**B**) *C. perfringens* CVCC 2032 treated without BMGlv2, 10.0 Kx and 20.0 Kx. (**C**,**D**) *C. perfringens* CVCC 2032 treated with BMGlv2 at MIC for 2 h, 10.0 Kx and 20.0 Kx.

SEM images of *C. perfringens* CVCC 2032 treated with BMGlv2 at its MIC showed that BMGlv2 significantly caused bacterial membrane damage. Compared with the control, the tested bacterial cells exposed to BMGlv2 exhibited an altered morphology with breakage and distortion (Figure 6A–D). The images also demonstrated that cells exposed to BMGlv2 secreted many intracellular lysates, which confirmed that the cell membranes of these bacteria were destroyed.

## 3. Discussion

AMPs are natural products that are potential substitutes for antibiotics and are active against pathogenic bacteria. Compared with antibiotics, the most important advantage of AMPs is that they do not easily produce drug-resistant organisms, which not only contributes to the sustainable development of agriculture, but also greatly ensures the food safety of agricultural products. Bacterial diarrhea is considered one of the common forms of diarrhea in piglet breeding, resulting in a low survival rate and poor immune performance in piglets. Among the pathogenic bacteria associated with piglet diarrhea, *E. coli*, *Salmonella*, and *C. perfringens* are considered as the largest causes of industrial loss. At present, many countries including China have legislated to prohibit the addition of antibiotics in feed. Therefore, it is important to explore novel AMPs that can effectively inactivate piglet diarrhea-related pathogens.

Currently, *E. coli* is the most common host for the heterologous expression of AMPs. The challenges of the *E. coli* expression system, such as the potential toxicity of endotoxins from the host cell wall, the damage AMPs cause to host cells which affects the fermentation density, the inability to express AMPs extracellularly, and the unsafety from the inducer IPTG in the process of fermentation, greatly limit the industrial application of AMPs [34]. As a GRAS microbial strain, the filamentous fungus *T. reesei* is gradually being recognized as a valuable microbial host. It shows strong protein secretion ability [35], and its complex extracellular polymeric substance can prevent extracellular secretion proteins from damaging fungal cells themselves [36]. Furthermore, *T. reesei* has native cellulase activity that helps to improve the digestion efficiency of cellulose in feeds [33]. The unique advantage of the expression of AMPs in *T. reesei* compared with the usually used host strains such as *E. coli* and *Pichia pastoris* is reflected in it being more conducive to the digestion and absorption of the nutrients by animals. *T. reesei* has been applied for the expression of cellulase [37], glyoxal oxidase [35], and cellobiohydrolase [38], but there are few studies on the expression of AMPs. As a promising host strain, the values of *T. reesei* including that there is no need for an inducer, it can be safely added into feed directly, and its native cellulase activity means there is more potential for it to be applied in the feed industry. The heterologous expression of AMPs in *T. reesei* provides an important support for its application as a feed additive in agriculture.

Gloverin is one of the glycine-rich AMPs and it was first identified in *Hyposphora cecropia* showing strong antimicrobial activity against *E. coli* D21f2 [39]. In the silkworm *Bombyx mori*, four gloverins (gloverin1-4) were identified according to their non-homologous gene sequences [40]. Among them, the activity of Gloverin2 could not be affected by high temperatures and acid treatments, which facilitates it withstanding the extreme environments of feed industrial processing [30,31]. Previous studies have overexpressed the gloverin2 in the silk gland to produce the silk with antimicrobial activity, which can expand the applications of silk in the biomedical industry [41]. Gloverin2 was also expressed in the prokaryotic expression system and it showed significant activity in inhibiting the growth of Gram-negative bacteria, including *E. coli* JM109 and *P. putida*, by disrupting the cell membrane and the cell integrity [30]. At present, gloverin2 is rarely expressed in filamentous fungal systems, and its application values in the feed industry and in the activity against *C. perfringens* has not been explored. In this study, BMGlv2 was successfully expressed in *T. reesei*, which improved its safety compared with the *E. coli* expression system. Three common pathogenic bacteria causing piglet diarrhea were used to evaluate the antimicrobial activity of BMGlv2. The MICs of BMGlv2 against *E. coli* ATCC 25922 (43.75 μg/mL), *S. derby* ATCC 13076 (43.75 μg/mL), and *C. perfringens* CVCC 2032 (21.86 μg/mL) exhibited that it has great potential when applied in feed to relieve and prevent bacterial piglet diarrhea. Additionally, in this study, BMGlv2 did not exhibit the obvious sporicidal activity for *C. perfringens* CVCC 2032.

The three tested bacteria may have pathogenicity for both animals and humans. Additionally, it is urgent and necessary to develop the application of antimicrobial peptides as an alternative to antibiotics in the animal breeding industry to provide safer animal disease control measures, especially to solve the problems of piglet diarrhea. Therefore, the activity of BMGlv2 against the three tested bacteria associated with piglet diarrhea was mentioned and focused on in this study. In addition, compared with previous studies, the activity of BMGlv2 against *E. coli* in this study (MIC of 43.75 μg/mL) was superior to BMGlv2 when expressed in the prokaryotic expression system (10 μM, the reported concentration of BMGlv2 expressed in *E. coli* with obvious antimicrobial activity) [30]. It has been broadly accepted that *T. reesei* has unique advantages. including its safety with regard to recombinant proteins, superior protein secretion capability, strong protein post-translative modification ability, and low fermentation cost [32]. However, there are few studies on the expression of BMGlv2; thus, the full and complete comparison of the results and advantages could not be performed except for the safety of the expressed protein and the activity mentioned above. Based on this, BMGlv2 expressed in *T. reesei* and its antimicrobial activity against the three bacteria associated with piglet diarrhea expand the application of AMPs in the animal breeding industry.

Except for the antimicrobial activity, salt sensitivity, thermostability, digestive enzyme sensitivity, and bactericidal kinetics are important for the preparation, processing, pretreatment, and application of BMGlv2. As BMGlv2 is a cationic peptide that can interact with the negative charge on the cell membrane of pathogenic bacteria, its antimicrobial activity may be affected by the interference of cations [42,43,44]. The antimicrobial activity was not significantly affected in the presence of certain cations in this study, which was consistent with the previous reports [45,46]. BMGlv2 exhibited superior thermostability and pepsin stability (pH 2.0), but was more sensitive to trypsin (pH 8.0), which is similar to the results of previously reported AMPs [47,48,49,50]. In addition, BMGlv2 almost completely killed the tested pathogenic bacteria within 3 h of treatment, which is comparable to the pharmacodynamics of some antibiotics, indicating that it has great therapeutic potential [47,51].

AMPs usually cause hemolysis of animal red blood cells, which greatly limits their application [52]. Therefore, hemolytic activity is an important indicator for evaluating the performance of AMPs. In this study, BMGlv2 showed no hemolytic activity against erythrocytes at the effective antimicrobial concentration, which shows that it has no cytotoxicity and is safe as a feed additive. One possible explanation is that BMGlv2 has a delicate balance of cationicity and hydrophobicity to maintain the maximum antimicrobial potency with minimal toxicity to cells [45,53].

As the bacterial cell membrane contains phospholipids or hydroxylated phospholipids, the surface of the bacteria is usually negatively charged, which is beneficial for the combination with cationic AMPs [54]. Most AMPs exert their antimicrobial activity by acting on bacterial cell membranes, and after a certain concentration of AMPs are combined with the bacteria, their conformation will rearrange, thereby destroying the cell membrane and causing the bacteria to be killed or inhibited [45,46,55]. The changes in the conductivity and bacterial contents indicated the cell membrane of *C. perfringens* CVCC 2032 was destroyed. For Gram-negative bacteria, the previous study showed that the cell wall component lipopolysaccharides (LPSs) could induce the binding of BMGlv2 with the cell surface; then, the bacterial cells’ integrity was destroyed [30] and the bacterial contents were leaked out. This alteration in conductivity and bacterial contents might be related to the change in the membrane potential of the cell plasma membrane, and the interaction of the AMPs with the cell membrane will destroy the larger potential on the cell plasma membrane, thereby allowing electric charge to flow [55,56]. SEM images further confirmed that the cell membrane of *C. perfringens* CVCC 2032 was destroyed by treatment with BMGlv2, and the bacterial morphology was altered. In addition, the cell wall component LPSs of Gram-negative bacteria, which are exposed to the outside, could interact with BMGlv2 and induce cell rupture and death [30]. BMGlv2 absorbs on the surface of bacterial cells, and leads to the alteration of bacterial morphology; therefore, the death of bacteria may be a canonical process. The results verified that BMGlv2 exerts antimicrobial activity by acting on the cell membrane of bacteria, and the exploration of the mechanism of its inhibitory activity provides a basis for further development and research on BMGlv2.

## 4. Materials and Methods

### 4.1. Materials

*E. coli* DH5α was purchased from Sigma-Aldrich (St. Louis, MO, USA) and used to prepare expression plasmids. The expression host strain *T. reesei* Tu6 ATCC MYA-256 was obtained from the American Type Culture Collection (ATCC). The expression vector of *T. reesei* Tu6, constructed with reference to a previous report [33] and named PCBHG, was deposited in our laboratory. Of the tested bacterial strains *E. coli* ATCC 25922, *S. derby* ATCC 13076, and *C. perfringens* CVCC 2032, the first two were obtained from the ATCC, and the third from the China Veterinary Culture Collection Center (Beijing, China). The three bacteria were used to measure the antimicrobial activity of the expressed peptide BMGlv2. All chemicals and reagents used in this study were purchased from Sigma-Aldrich and were of analytical grade.

### 4.2. Construction of the Expression Vector PCBHG-BMGlv2

The amino acids of BMGlv2 were obtained online from the NCBI using the protein ID ANS56421.1. The gene encoding the mature protein was optimized based on the preference of the host codon using the software Vector NTI 13.5. Then, the gene with the GenBank accession number of OL331022 was synthesized and ligated into a general expression vector by the Ruijie Biological Engineering Company (Shanghai, China). Subsequently, the gene was amplified using a polymerase chain reaction (PCR) and primers with homology arm sequences at both ends of the vector insertion site (forward primer F: 5′-TCTTGGCCACAGCTCGTGCTGAATTCATGAACTCTAACTTGTTTTACATTTTTG-3′; reverse primer R: 5′-TCAGGCTTTCGCCACGGAGCGCGGCCGCTTATTACCAAT-3′). The expression vector PCBHG was linearized by PCR using the Phanta^®^ Super-Fidelity DNA Polymerase (Vazyme Biotech Co., Ltd., China), and the linearization primers used were the sequences at both ends of the cloning site of the vector (forward primer F: 5′-CATCATCATCATCATCATTAGTAAGCTCCG-3′; reverse primer R: 5′-AGCACGAGCTGTGGCCAAGAAG-3′). The cloning site of PCBHG was located between the promoter and terminator, and the vector was linearized by PCR to facilitate the insertion of the target gene. The target gene, which was purified using the E.Z.N.A^®^ Cycle Pure Kit D6492, was ligated to the linearized vector that was purified, using the same kit, and reacted at 37 °C for 30 min using Exnase II. Then, the integrated vector was transformed into *E. coli* DH5α, and the transformants were grown on Luria–Bertani (LB) solid plates containing 100 μg/mL Zeocin. Colonies were then selected and transferred to a new LB solid plate containing 100 μg/mL Zeocin. After sequencing verification and alignment of the selected colonies by RuiBiotech Co., Ltd. (Beijing, China), the expression vector PCBHG-BMGlv2 was successfully constructed.

### 4.3. Transformation and Expression of PCBHG-BMGlv2 in T. reesei and Purification of BMGlv2

The polyethylene glycol-mediated transformation of protoplasts was performed as previously described [57]. *T. reesei* Tu6 was cultured on potato dextrose agar medium containing 1% uracil at 30 °C for 6 d, and protoplasts were prepared by enzymatic lysis with a lyase at 80 rpm and 30 °C for 2 h, which were used as transformation hosts. The number of protoplasts was at least 10^7^ per 200 μL of lysis solution, and their normal growth state was observed under a microscope. Then, the expression vector was transferred into the protoplasts, incubated on ice for 25 min, spread evenly in the culture medium without uracil, and cultured at 30 °C for 6 d. The growth state and number of transformants on the transformant solid medium plates without uracil were monitored continuously during the growth period.

After the transformants grew, rounded transformants were selected for positive verification. The genomic DNA of the selected transformants was extracted using the E.Z.N.A^®^ Fungal DNA Kit D3390 after the mycelia were ground with liquid nitrogen. A PCR was performed to verify the target gene, and a positive transformant was obtained. The transformants with adequate spores were transferred to the MM liquid medium at 180 rpm and 30 °C for 6 d to express BMGlv2. The fermentation supernatant was collected by centrifugation at 6000 rpm for 10 min. Sodium dodecyl sulfate-polyacrylamide gel electrophoresis (SDS-PAGE) was used to detect the expression of the target proteins in the fermentation broth.

The recombinant BMGlv2 was purified using a Ni-sepharose 6FF column (GE Healthcare, United States) as previously described [58]. Briefly, 50 mL of the fermentation mixture was centrifuged at 10,000× *g* for 10 min to collect the supernatant, and the collected supernatant was filtered through a 0.45 µm membrane filter (OBT, Beijing, China). Then, the supernatant was treated with solid ammonium sulfate and kept at 4 °C overnight. The sediment was redissolved and dialyzed in distilled water for desalination. The further purification was operated by injecting the sample into a Ni-sepharose 6FF column and the His-tagged target peptide was eluted with imidazole (50–150 mM). The purified fraction was analyzed by SDS-PAGE. The antimicrobial activity (AU/mL) of purified BMGlv2 was calculated to evaluate the purified peptide as previously described [59,60]; the equation was referenced as follows: AU/mL = (diameter of the zone of clearance (mm) * 1000)/ volume taken in the well (μL). Here, the indicated bacterial strain involved in the assessment of the antibacterial activity of purified BMGlv2 was *C. perfringens* CVCC 2032. All assays were performed in triplicate.

### 4.4. Measurement of Antimicrobial Activity of BMGlv2

The antimicrobial activity of purified BMGlv2 was further measured to analyze its minimum inhibitory concentration (MIC) and minimum bactericidal concentration (MBC) values using the microbroth dilution method, as previously described by the Clinical and Laboratory Standards Institute, against the three tested bacteria in cultivation, including a Gram-positive bacterial strain (*C. perfringens* CVCC 2032) and two Gram-negative strains (*E. coli* ATCC 25922 and *S. derby* ATCC 13076). Briefly, 50 μL of the antimicrobial peptide solution was added to 96-well plates containing 50 μL of liquid medium (LB used for *E. coli* and *S. derby*, brain heart infusion (BHI) used for *C. perfringens*) to the final concentration of the purified peptide of 175 μg/mL, then was two-fold diluted with a liquid medium to different concentration. *E. coli* ATCC 25922 and *S. derby* ATCC 13076 were incubated in LB liquid medium at 37 °C and 180 rpm. *C. perfringens* CVCC 2032 was inoculated into 20 mL of BHI liquid medium containing 1 g/L of cysteine hydrochloride, used as the reducing agent, which was placed in advance in the 50 mL anaerobic bottles and was continuously filled with nitrogen for almost 20 min to maintain the strictly anaerobic environment before sealing with butyl rubber stoppers and an aluminum crimp. Then, it was grown in an anaerobic incubator at 37 °C. After the mature bacterial suspension was diluted to 10^5^ CFU/mL with the culture medium, 50 μL of it was added to the double-diluted antimicrobial peptide solution and incubated at 37 °C for 18 h. Ampicillin was used as the positive control for the concentrations from 60 μg/mL to 1.87 μg/mL in the process of measurements. The MIC was determined as the lowest concentration at which no visible bacterial growth was observed, and the MBC was determined as the lowest concentration that inhibited 99.9% of the tested bacterial growth [61,62].

### 4.5. Sensitivity Analysis to Temperature and Salinity

The three tested bacterial strains acted as indicator bacteria to determine the temperature and salt sensitivity of BMGlv2. The salt sensitivity of BMGlv2 was measured in different salts to assess their effect on the MICs of BMGlv2. The AMP solution was continuously double diluted in a 96-well plate using different salts at their physiological concentrations, including NaCl (150 mM), KCl (4.5 mM), NH_4_Cl (6.0 μM), ZnCl_2_ (8.0 μM), MgCl_2_ (1.0 mM), and FeCl_3_ (4.0 μM). After the diluted bacteria (10^5^ CFU/mL) were added and mixed with the AMP solution, the 96-well plate was incubated at 37 °C for 18 h, and the MICs were recorded. The thermal stability of BMGlv2 was determined after incubation in boiling water (100 °C) for 5, 10, 15, 20, 25, and 30 min. The treated samples were added to the diluted bacteria (10^5^ CFU/mL), and the mixed samples were incubated at 37 °C for 18 h. Optical density (OD) was measured at 600 nm.

### 4.6. Evaluation of Resistance to Digestive Enzymes

Pepsin and trypsin were used to perform a proteolytic digestion resistance assay using BMGlv2. Pepsin was diluted to 3000 U/mg with Gly-HCl buffer at pH 2.0, and trypsin was diluted to 250 U/mg with Tris-HCl buffer at pH 8.0. The BMGlv2 solution was diluted to the MIC with pepsin (3000 U/mg, pH 2.0) and trypsin (250 U/mg, pH 8.0). The diluted BMGlv2 solution was then incubated at 37 °C for different lengths of time, ranging from 30 to 180 min. Then, 50 µL of each sample was added to the diluted bacteria (10^5^ CFU/mL), and the mixture was incubated at 37 °C for 18 h. The OD_600 nm_ was measured, and the relative antimicrobial activity under different treatments was calculated.

### 4.7. Bactericidal Kinetic Assays

The initial concentrations of the indicator bacteria (*E. coli* ATCC 25922, *S. derby* ATCC 13076, and *C. perfringens* CVCC 2032) were diluted to 10^5^ CFU/mL using a liquid culture; 50 μL of these were added to the BMGlv2 solution at the MIC, and the mixed samples were incubated at 37 °C. Samples were collected at different times (30, 60, 90, 120, and 180 min) and evenly coated onto the LB solid medium plate (used for *E. coli* and *S. derby*) and the BHI solid medium plate (used for *C. perfringens*). After incubation at 37 °C for 18 h, the visible colonies were counted. Treatment of ampicillin (AMP) was used as the positive control and the bacterial solution with no treatment was used as the negative control.

### 4.8. Measurement of Hemolytic Activity

Fresh rabbit red blood cells (rRBCs), used to determine the hemolytic activity of BMGlv2, were washed and resuspended in phosphate-buffered saline (PBS) three times to collect more than 1% (*v*/*v*) of erythrocytes; then, 50 μL of rRBC solution was mixed with 50 μL of continuous double-diluted BMGlv2 solution. After 1 h of incubation at 37 °C, the mixture was centrifuged at 1200 rpm for 10 min, and the absorbance of the supernatant was measured at 570 nm. The positive control was 0.1% Triton X-100, which had 100% hemolysis values, and the PBS served as a negative control. The hemolysis values (%) were defined according to the equation: (OD_570_ of BMGlv2 solution − OD_570_ of PBS)/(OD_570_ of 0.1% Triton X-100 − OD_570_ of PBS) × 100%.

### 4.9. Antibacterial Mechanism against C. perfringens

The bacterial protein and DNA leakage and changes in the conductivity of the solution during bacterial growth were measured as previously described [63,64,65,66,67]. Furthermore, *C. perfringens* that grew to the logarithmic period were diluted to 10^8^ CFU/mL with liquid culture as the bacteria to be tested. The BMGlv2 solution was diluted to the MIC and mixed with the diluted bacteria. The mixture was incubated at 37 °C for 24 h, and the samples were obtained every 2 h to analyze protein and DNA leakage and changes in the conductivity of the solution. Protein leakage was measured using the Coomassie bright blue G-250 colorimetric method [65], and DNA leakage was determined at OD_260 nm_ [63,66,68]. The conductivity of the solution was measured using a conductometer. To better understand the bacterial time-killing of BMGlv2 against *C. perfringens*, the initial concentrations of the indicator bacteria (*C. perfringens* CVCC 2032) were diluted to 10^5^ CFU/mL using the BHI liquid medium; 50 μL of these were added to the BMGlv2 solution at the MIC, and the mixed samples were incubated at 37 °C for 24 h. They were collected every 2 h and evenly coated onto BHI solid medium plates. After incubation at 37 °C for 18 h, the visible colonies were counted, indicating the remaining live bacterial cells after the treatment of BMGlv2.

### 4.10. SEM Observations

Next, *C. perfringens* CVCC 2032 bacterial cells in the mid-log phase were suspended in PBS at a concentration of 10^7^ CFU/mL and then incubated with BMGlv2 at its MIC at 37 °C for 2 h. Furthermore, 10^7^ CFU/mL of bacterial cells in the absence of BMGlv2 was used as the negative control. After incubation, the samples were washed twice with PBS and fixed in 2.5% glutaraldehyde solution at 4 °C overnight. The observation of samples using SEM was operated in Qingdao University Medical College (China) with acceleration voltages of 10 kV and 20 kV after the corresponding processing [69].

### 4.11. Statistical Analysis

All experiments were independently conducted three times, and the results were given as the mean ± standard deviation (SD). The results and data were statistically analyzed using an analysis of variance (*t*-test) with the IBM SPSS Statistics 26 software. Differences were considered significant at a threshold of *p* < 0.05.

## 5. Conclusions

In conclusion, BMGlv2 derived from *B. mandarina* was heterologously expressed in *T. reesei* Tu6, a potential engineered strain developed because of its unique safety and cellulase activity compared with the usually used host strains. BMGlv2 shows desirable antimicrobial activity against three indicator bacteria associated with piglet diarrhea (*E. coli*, *S. derby*, and *C. perfringens*). The thermostability of BMGlv2 provides an advantage for the preparation of feed additives during feedstuff processing at high temperatures. BMGlv2 maintains its antimicrobial activity in the presence of pepsin and at pH 2.0, indicating that BMGlv2 has the advantage of passing through the stomach and exerting its activity in the intestine. Furthermore, it has low hemolytic activity against rRBCs, suggesting that BMGlv2 has the potential to be applied as a safe and low-cytotoxicity feed additive. Therefore, the antimicrobial activity, thermostability, acid and pepsin tolerance, and low hemolytic activity of BMGlv2 strongly support and justify its application in the feed industry. Its expression in *T. reesei* further lays a foundation for the safety of its application as a feed additive.

## Figures and Tables

**Table 1:** Purification of recombinant of BMGlv2.

Sample	Volume (mL)	Activity (AU/mL)	Total Protein (mg)	Total Activity (AU)	Specific Activity (AU/mg)	Yield (%)	Purification (Fold)
Culture supernatant	50	150	9.3	7500	806.5	100	1
Purified BMGlv2	14	292	1.6	4088	2555	54.5	3.2

**Table 2:** MIC values and MBC values of BMGlv2.

Bacteria	MIC (μg/mL)		MBC (μg/mL)	
	BMGlv2	Ampicillin	BMGlv2	Ampicillin
Gram-negative				
*E. coli* ATCC 25922	43.75	3.75	43.75	3.75
*S. derby* ATCC 13076	43.75	7.50	43.75	7.50
Gram-positive				
*C. perfringens* CVCC 2032	21.86	3.75	43.75	3.75

**Table 3:** MIC values (μg/mL) of BMGlv2 in the presence of salts.

Strains	Control	NaCl (150 mM)	KCl (4.5 mM)	NH_4_Cl (6 μM)	MgCl_2_ (1 mM)	ZnCl_2_ (8 μM)	FeCl_3_ (4 μM)
*E. coli* ATCC 25922	43.75	21.86	43.75	21.86	43.75	21.86	21.86
*S. derby* ATCC 13076	43.75	43.75	43.75	43.75	43.75	43.75	43.75
*C. perfringens* CVCC 2032	21.86	10.93	21.86	21.86	10.93	10.93	10.93

## Data Availability

All data generated or analyzed during this study are included in this article.

## Supplementary Materials

The following supporting information can be downloaded at: https://www.mdpi.com/article/10.3390/ijms231810291/s1.
