# Supplementary material for: Characterization of Recombinant Antimicrobial Peptide BMGlv2 Heterologously Expressed in Trichoderma reesei
Source: Int J Mol Sci. 2022 Sep 7;23(18):10291. doi: 10.3390/ijms231810291 (PMC9499586; doi:10.3390/ijms231810291)
Supplement: Supplementary file 1 [file ijms-23-10291-s001.zip › ijms-1865425-supplementary.pdf]

Supplemental materials

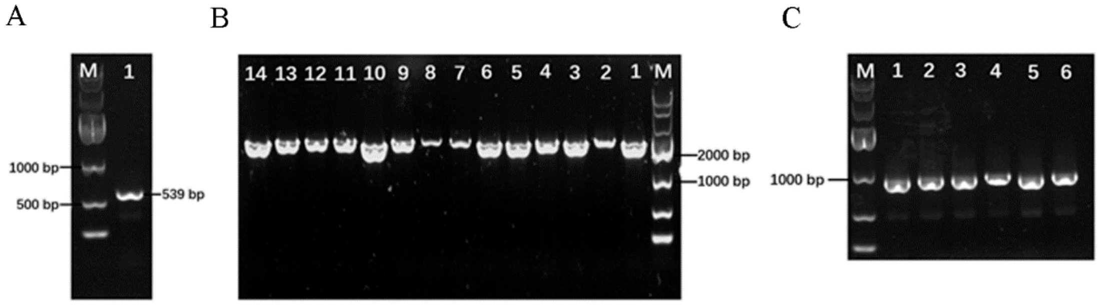

Figure S1. Results of agarose gel electrophoresis. (A) Amplification analysis of the genes encoding BMGlv2. Lane M, DNA marker; Lane 1, PCR product of genes encoding BMGlv2. (B) Colony PCR products for verification of DH5α transformants of PCBHG-BMGlv2. Lane M, DNA marker; Lane 1-14, Positive colony PCR products of DH5α transformants of PCBHG-BMGlv2, including genes encoding BMGlv2 and partial expression vector (about 2000 bp). (C) PCR products for verification of genomes of BMGlv2 transformants in *T. reesei* Tu6. Lane M, DNA marker; lane 1-6, Positive PCR products of genomes of BMGlv2 transformants in *T. reesei* Tu6, including genes encoding BMGlv2 and partial expression vector (about 800 bp).
